# Dental Economics

**Published:** 1913-08-15

**Authors:** 


### DENTAL ECONOMICS.

Because of the awakened discussions concerning business and business methods in dentistry, many of our more conservative men fear lest the profession be prostituted in consequence. There should be no danger of that and a free discussion of dental economic subjects should result only in good not only to the profession itself and its personnel, but also to the public. If we do not lose sight of the real purpose of the profession and of its higher ideal and deliberately seek the methods of the charlatan and grafter, good will come from a proper attention to business methods. We are not called upon to render charity through negligence, stupidity or indolence where charity is not required or sought and we should be paid fairly and properly for our services. This in itself can not in any way interfere with our cherished ideas concerning ethics, and will place us in a much better position to bestow gratuitous services where such services are called for. Our obligations to properly care for our families and properly assume the responsibilities of citizenship are large, and we do not in any way discharge them if we are slovenly and lax in our affairs of business. This is no plea for higher prices, though they are many times indicated, but it is a suggestion that we give more attention to what it costs us to produce our work; more attention to the close collection of our accounts, and much more attention to the proper husbandry and investing of such moneys as come our way. It is not contemplated in the most altruistic code imagineable that one should come in his last days, when he is old and worn out, to be a pensioner upon the bounty of others. So that the present discussions, if they are taken in the proper spirit, and for the good they contain, must be of benefit to all. Editorial in *Western Dental Journal.*

